# Supplementary material for: Surveillance of Antimicrobial Resistance of Escherichia coli Isolates from Intestinal Contents of Dairy and Veal Calves in the Veneto Region, Northeaster Italy
Source: Animals (Basel). 2024 May 10;14(10):1429. doi: 10.3390/ani14101429 (PMC11117218; doi:10.3390/ani14101429)
Supplement: Supplementary file 1 [file animals-14-01429-s001.zip › animals-2974837-supplementary.pdf]

Figure S1. Temporal distribution of the minimum inhibitory concentration values (MIC, μg/mL) of ampicillin (AMP), colistin (COL), enrofloxacin (ENR), florfenicol (FLO), flumequine (FLQ), gentamicin (GEN), paromomycin (PRM), tetracycline (TET), and sulfamethoxazole/trimethoprim (SX-T).

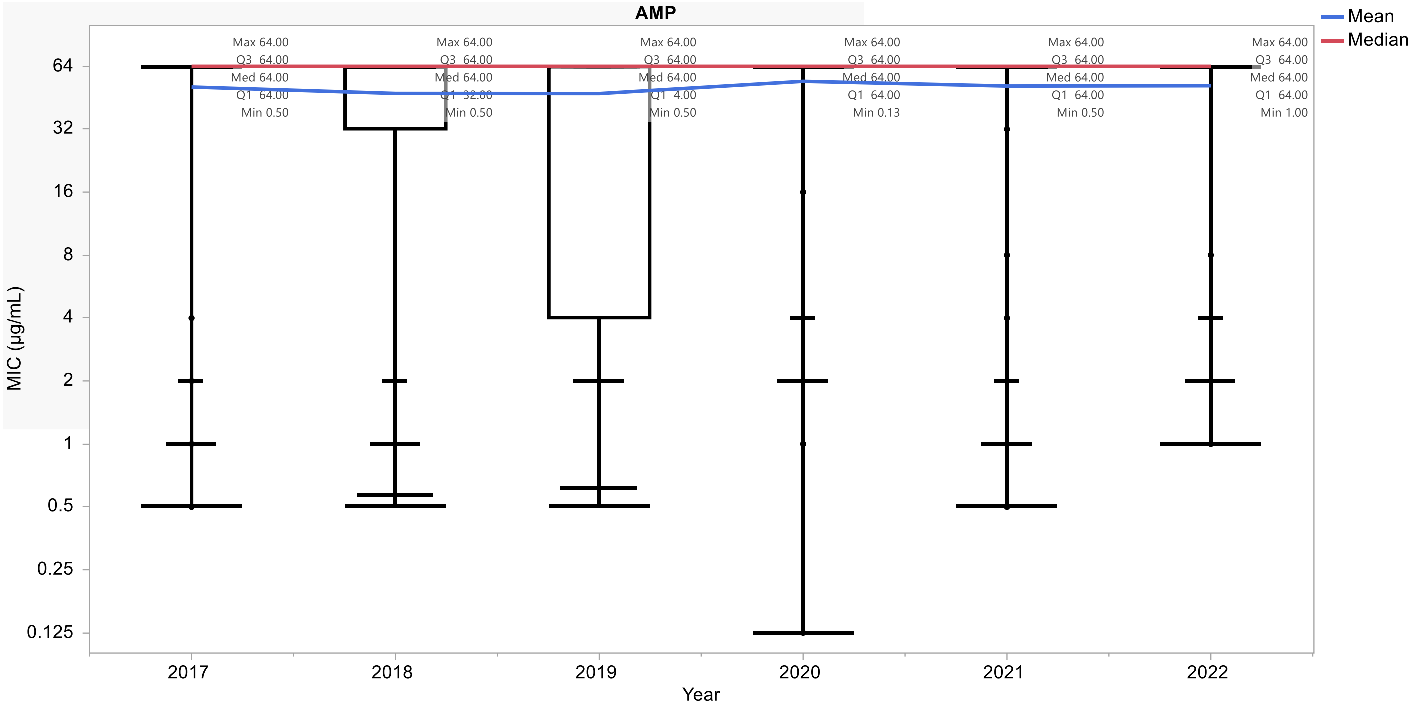

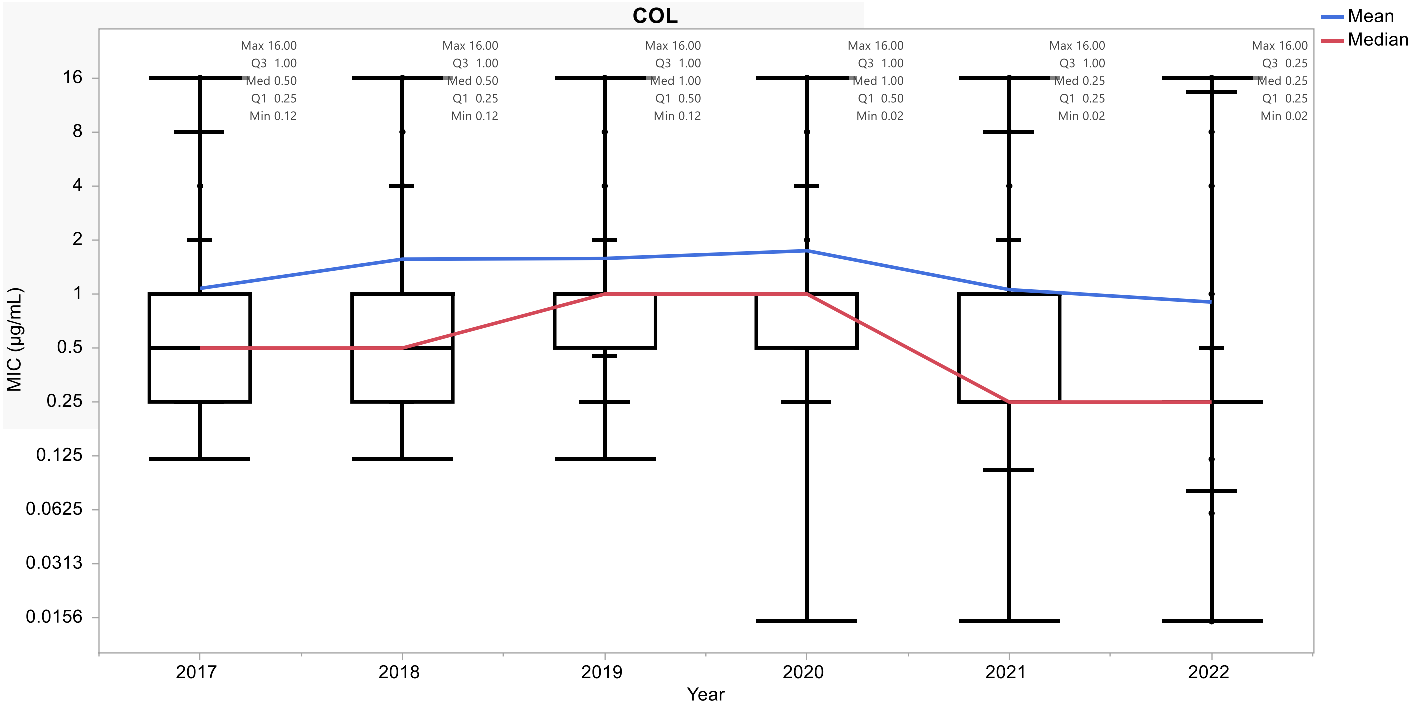

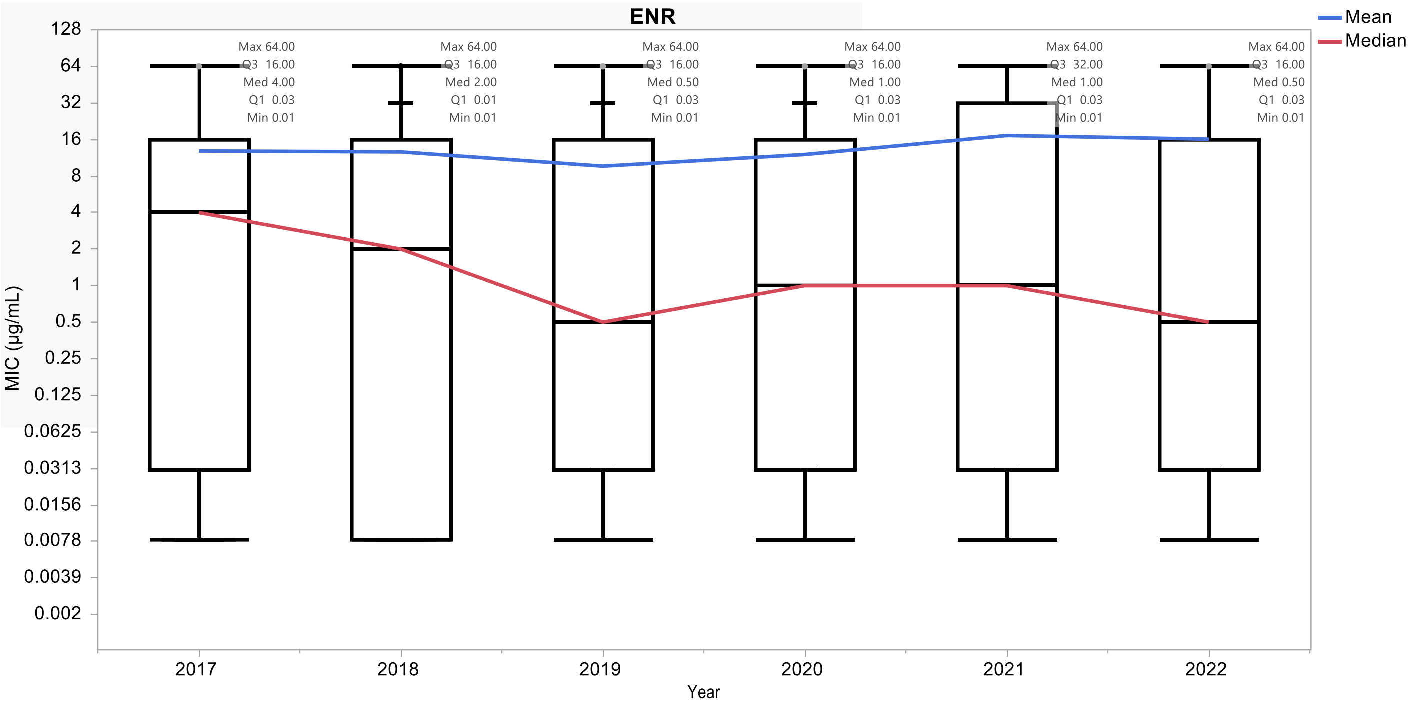
ENR
Mean
Median
MIC (µg/mL)
128
64
32
16
8
4
2
1
0.5
0.25
0.125
0.0625
0.0313
0.0156
0.0078
0.0039
0.002
Max 64.00 Q3 16.00 Med 4.00 Q1 0.03 Min 0.01
Max 64.00 Q3 16.00 Med 2.00 Q1 0.01 Min 0.01
Max 64.00 Q3 16.00 Med 0.50 Q1 0.03 Min 0.01
Max 64.00 Q3 16.00 Med 1.00 Q1 0.03 Min 0.01
Max 64.00 Q3 32.00 Med 1.00 Q1 0.03 Min 0.01
Max 64.00 Q3 16.00 Med 0.50 Q1 0.03 Min 0.01
2017
2018
2019
2020
2021
2022
Year

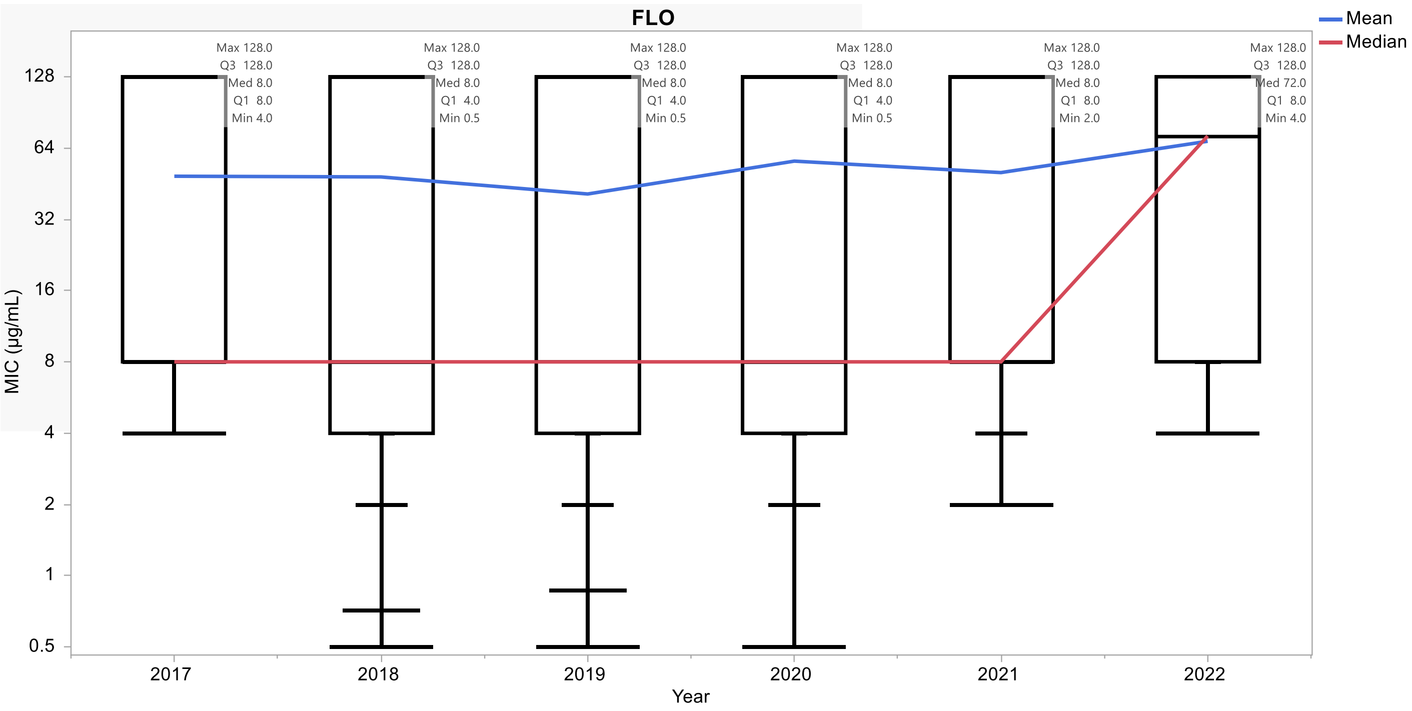
FLO
Mean
Median
MIC (µg/mL)
128
64
32
16
8
4
2
1
0.5
Max 128.0 Q3 128.0 Med 8.0 Q1 8.0 Min 4.0
Max 128.0 Q3 128.0 Med 8.0 Q1 4.0 Min 0.5
Max 128.0 Q3 128.0 Med 8.0 Q1 4.0 Min 0.5
Max 128.0 Q3 128.0 Med 8.0 Q1 4.0 Min 0.5
Max 128.0 Q3 128.0 Med 8.0 Q1 8.0 Min 2.0
Max 128.0 Q3 128.0 Med 72.0 Q1 8.0 Min 4.0
2017
2018
2019
2020
2021
2022
Year

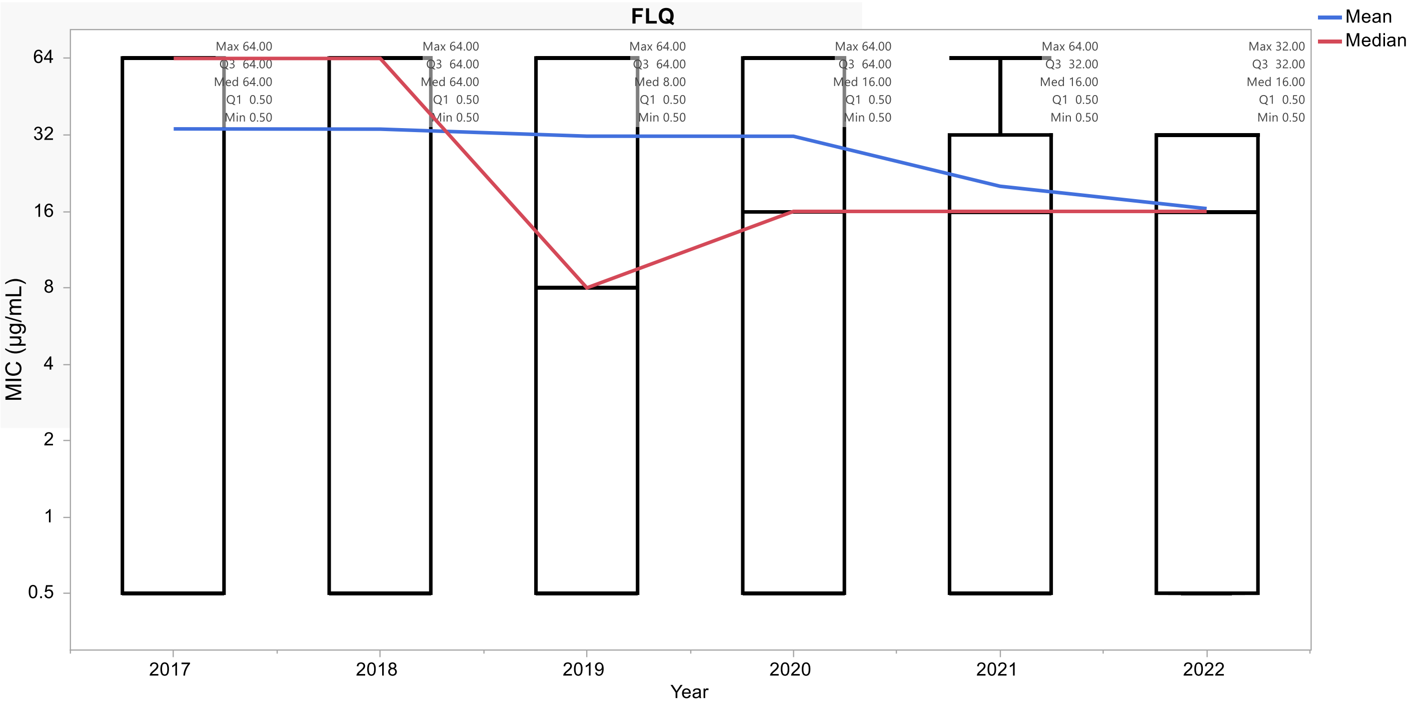
FLQ
Mean
Median
MIC (µg/mL)
Year
2017
2018
2019
2020
2021
2022
Max 64.00 Q3 64.00 Med 64.00 Q1 0.50 Min 0.50
Max 64.00 Q3 64.00 Med 64.00 Q1 0.50 Min 0.50
Max 64.00 Q3 64.00 Med 8.00 Q1 0.50 Min 0.50
Max 64.00 Q3 64.00 Med 16.00 Q1 0.50 Min 0.50
Max 64.00 Q3 32.00 Med 16.00 Q1 0.50 Min 0.50
Max 32.00 Q3 32.00 Med 16.00 Q1 0.50 Min 0.50

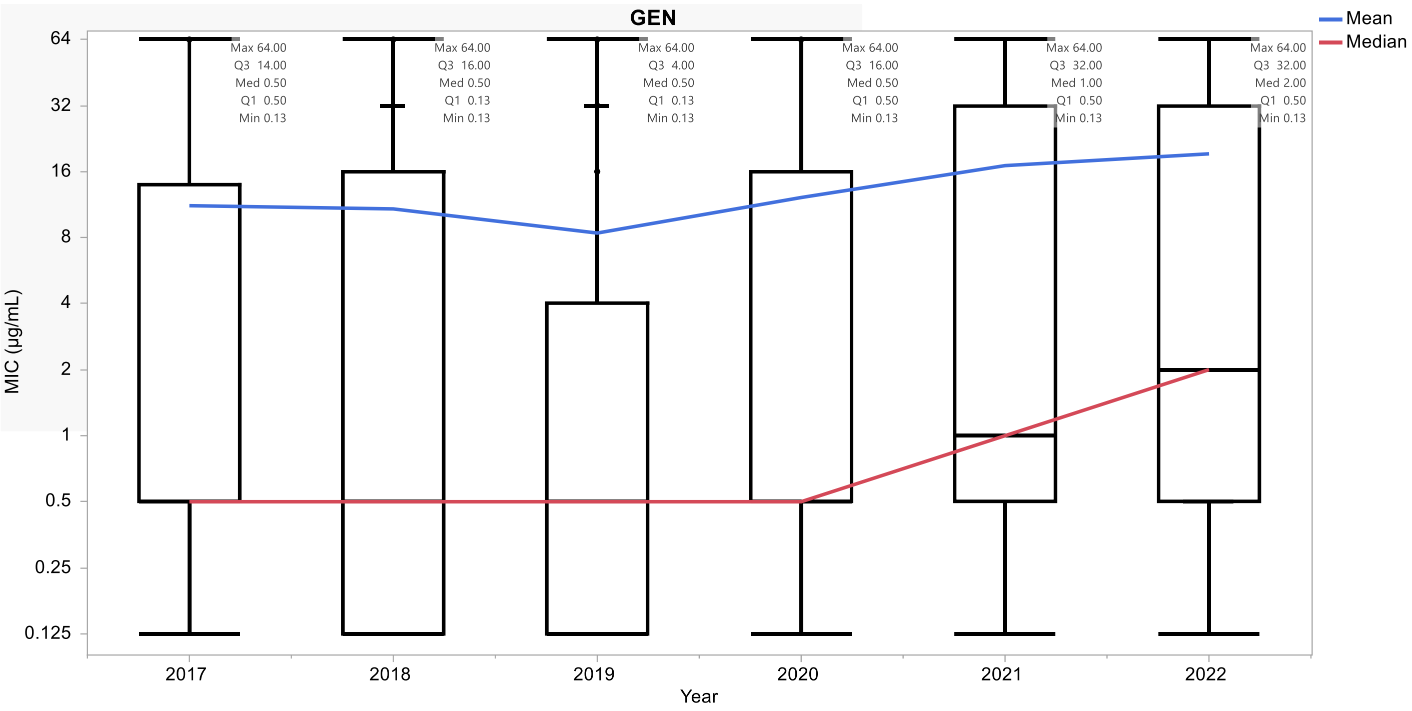
GEN
Mean
Median
MIC (µg/mL)
Year
2017
2018
2019
2020
2021
2022
Max 64.00 Q3 14.00 Med 0.50 Q1 0.50 Min 0.13
Max 64.00 Q3 16.00 Med 0.50 Q1 0.13 Min 0.13
Max 64.00 Q3 4.00 Med 0.50 Q1 0.13 Min 0.13
Max 64.00 Q3 16.00 Med 0.50 Q1 0.50 Min 0.13
Max 64.00 Q3 32.00 Med 1.00 Q1 0.50 Min 0.13
Max 64.00 Q3 32.00 Med 2.00 Q1 0.50 Min 0.13

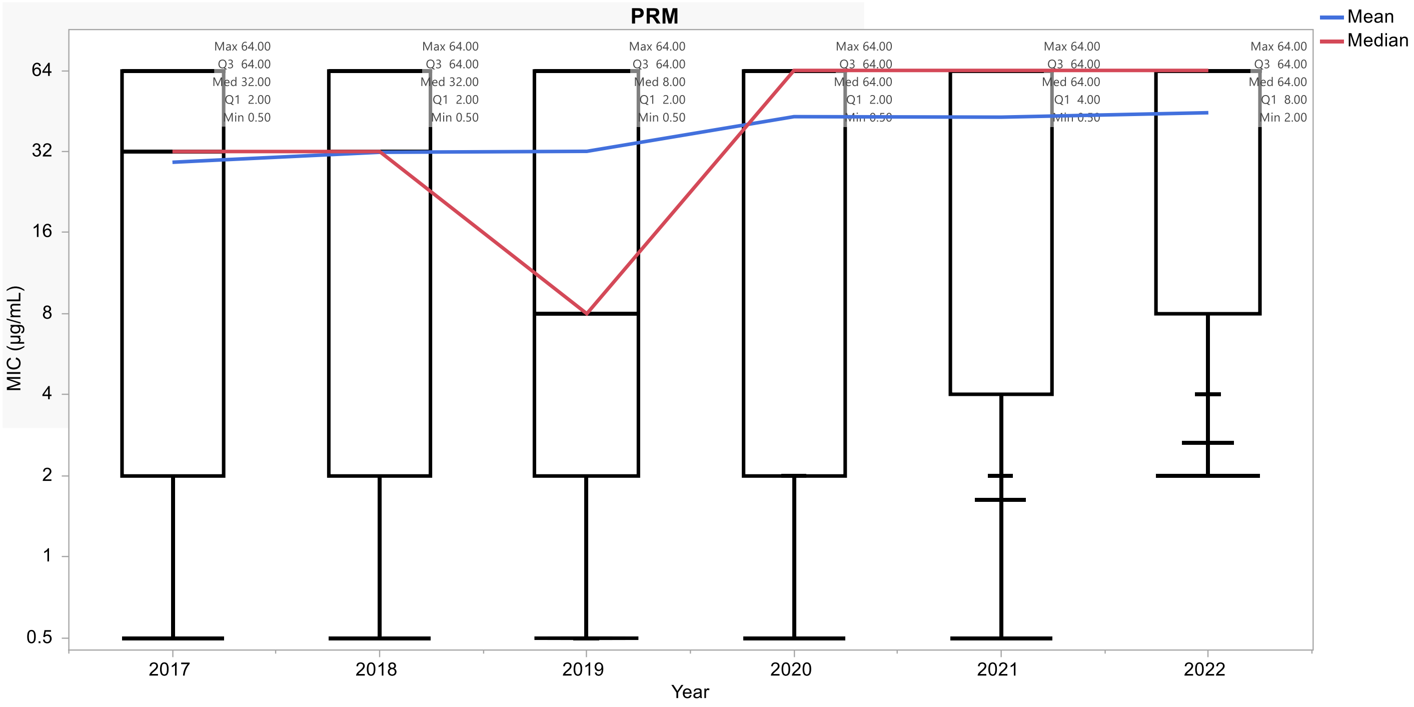
PRM
Mean
Median
MIC (µg/mL)
Year
2017
2018
2019
2020
2021
2022
Max 64.00 Q3 64.00 Med 32.00 Q1 2.00 Min 0.50
Max 64.00 Q3 64.00 Med 32.00 Q1 2.00 Min 0.50
Max 64.00 Q3 64.00 Med 8.00 Q1 2.00 Min 0.50
Max 64.00 Q3 64.00 Med 64.00 Q1 2.00 Min 0.50
Max 64.00 Q3 64.00 Med 64.00 Q1 4.00 Min 0.50
Max 64.00 Q3 64.00 Med 64.00 Q1 8.00 Min 2.00

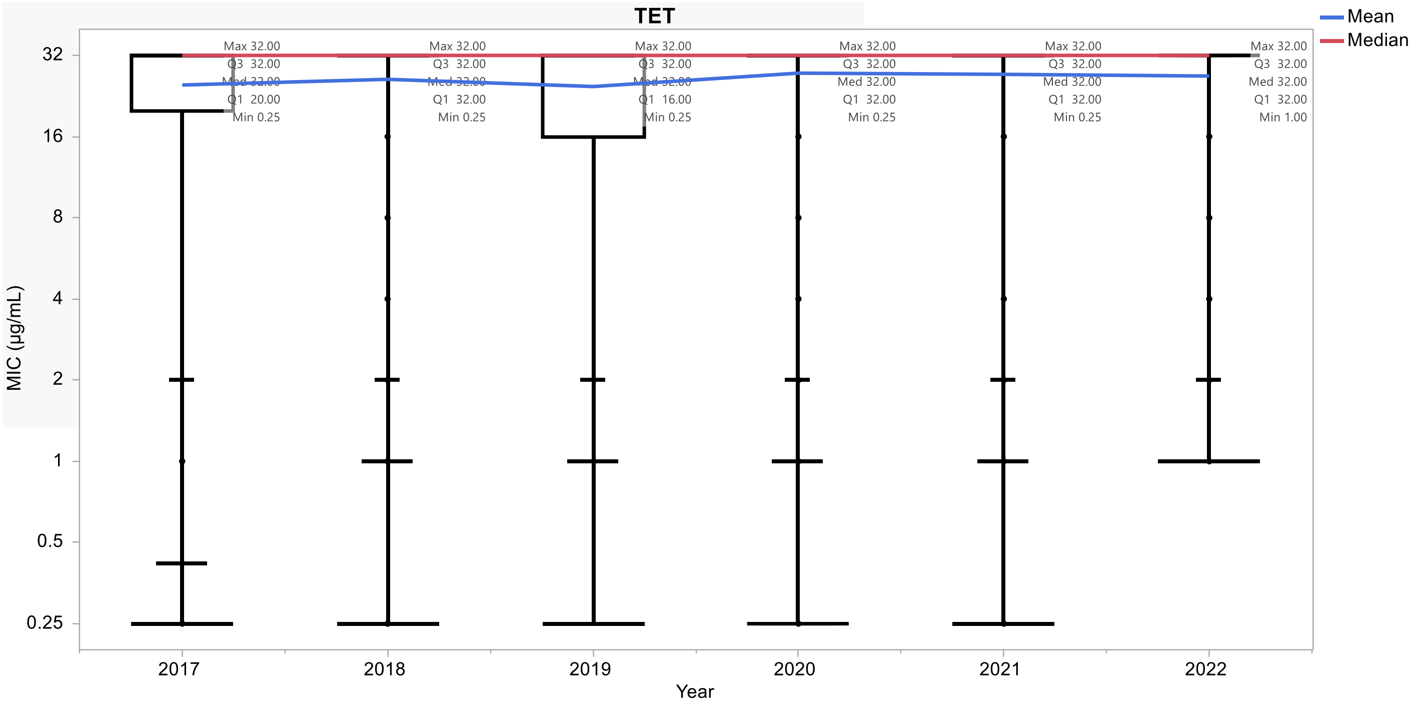
TET
Mean
Median
MIC (µg/mL)
Year
2017
2018
2019
2020
2021
2022
Max 32.00 Q3 32.00 Med 32.00 Q1 20.00 Min 0.25
Max 32.00 Q3 32.00 Med 32.00 Q1 32.00 Min 0.25
Max 32.00 Q3 32.00 Med 32.00 Q1 16.00 Min 0.25
Max 32.00 Q3 32.00 Med 32.00 Q1 32.00 Min 0.25
Max 32.00 Q3 32.00 Med 32.00 Q1 32.00 Min 0.25
Max 32.00 Q3 32.00 Med 32.00 Q1 32.00 Min 1.00
